# Effects of Air Pollution on Heart Rate Variability: The VA Normative Aging Study

**DOI:** 10.1289/ehp.7447

**Published:** 2004-12-06

**Authors:** Sung Kyun Park, Marie S. O’Neill, Pantel S. Vokonas, David Sparrow, Joel Schwartz

**Affiliations:** ^1^Department of Environmental Health, Harvard School of Public Health, Boston, Massachusetts, USA; ^2^VA Normative Aging Study, Veterans Affairs Boston Healthcare System and the Department of Medicine, Boston University School of Medicine, Boston, Massachusetts, USA

**Keywords:** air pollution, diabetes, heart rate variability, hypertension, ischemic heart disease, ozone, PM_2.5_

## Abstract

Reduced heart rate variability (HRV), a marker of poor cardiac autonomic function, has been associated with air pollution, especially fine particulate matter [< 2.5 μm in aerodynamic diameter (PM_2.5_)]. We examined the relationship between HRV [standard deviation of normal-to-normal intervals (SDNN), power in high frequency (HF) and low frequency (LF), and LF:HF ratio] and ambient air pollutants in 497 men from the Normative Aging Study in greater Boston, Massachusetts, seen between November 2000 and October 2003. We examined 4-hr, 24-hr, and 48-hr moving averages of air pollution (PM_2.5_, particle number concentration, black carbon, ozone, nitrogen dioxide, sulfur dioxide, carbon monoxide). Controlling for potential confounders, HF decreased 20.8% [95% confidence interval (CI), 4.6–34.2%] and LF:HF ratio increased 18.6% (95% CI, 4.1–35.2%) per SD (8 μg/m^3^) increase in 48-hr PM_2.5_. LF was reduced by 11.5% (95% CI, 0.4–21.3%) per SD (13 ppb) increment in 4-hr O_3_. The associations between HRV and PM_2.5_ and O_3_ were stronger in people with ischemic heart disease (IHD) and hypertension. The associations observed between SDNN and LF and PM_2.5_ were stronger in people with diabetes. People using calcium-channel blockers and beta-blockers had lower associations between O_3_ and PM_2.5_ with LF. No effect modification by other cardiac medications was found. Exposures to PM_2.5_ and O_3_ are associated with decreased HRV, and history of IHD, hypertension, and diabetes may confer susceptibility to autonomic dysfunction by air pollution.

Short- and long-term exposure to air pollution has been associated with increased cardiovascular mortality and morbidity (Pope et al. 2002; Samet et al. 2000; Schwartz 1999), and individuals with underlying cardiovascular disease, including heart failure, arrhythmia, or diabetes, are at greater risk (Bateson and Schwartz 2004; Goldberg et al. 2001; Mann et al. 2002; Zanobetti and Schwartz 2002). Possible mechanisms for these associations include effects on the autonomic nervous system through direct reflexes from airways or through inflammatory response, chemical effects on ion channel function in myocardial cells, ischemic response in the myocardium, and inflammatory responses that trigger endothelial dysfunction, atherosclerosis, and thrombosis (Utell et al. 2002).

Heart rate variability (HRV) is a widely used noninvasive and quantitative marker of cardiac autonomic control. HRV reflects autonomic modulation of the rhythmic activity of the sinus node and is analyzed in the time or frequency domains (Task Force 1996). Sustained reductions of HRV have been associated with increased risk of mortality in middle-age and elderly subjects, in patients with diabetes, and in survivors of myocardial infarction and other cardiovascular diseases (Dekker et al. 1997; Gerritsen et al. 2001; Tapanainen et al. 2002; Tsuji et al. 1996). Air pollution, especially particulate matter < 2.5 μm in aero-dynamic diameter (PM_2.5_), has been associated with alterations in HRV (Creason et al. 2001; Devlin et al. 2003; Gold et al. 2000; Holguin et al. 2003; Liao et al. 1999, 2004; Magari et al. 2001, 2002; Pope et al. 1999, 2004). However, only two studies explored whether clinical conditions or other subject characteristics modified the association between air pollution and HRV (Holguin et al. 2003; Liao et al. 2004). Little has been reported to date on associations with particle components.

In this study we examine the relationship between alterations in HRV and ambient air pollutants among community residents. We also investigated modifying effects of hypertension, ischemic heart disease (IHD), diabetes, and use of commonly prescribed antihypertensive medications that increase cardiac vagal activity (Lampert et al. 2003; Tomiyama et al. 1998; Townend et al. 1995).

## Materials and Methods

### Study population.

The Normative Aging Study is a longitudinal study of aging established by the Veterans Administration in 1963, when 2,280 men from the Greater Boston area (21–81 years of age) confirmed to be free of known chronic medical conditions were enrolled (Bell et al. 1972). Participants were asked to return for examinations every 3–5 years. Among active cohort members, 603 persons were examined from 14 November 2000 through 30 October 2003.

Participants visited the study center in the morning, after an overnight fast and abstinence from smoking. Weight and height were measured to compute body mass index (BMI). With the subject seated, heart rate and systolic and diastolic blood pressures were measured by a physician. The mean of the left and right arm measurements was used. For this study, we defined mean arterial blood pressure (MAP) as diastolic pressure plus one-third of the difference between systolic and diastolic blood pressure. Subjects with diabetes were defined by a physician’s diagnosis of type 2 diabetes and/or use of a diabetes medication (e.g., oral hypoglycemic drug, metformin, or insulin). Hypertension was defined as systolic blood pressure of ≥140 mm Hg, diastolic blood pressure of ≥90 mm Hg, or reported use of hypertension medication. Cigarette smoking, alcohol consumption, and subjects’ use of medications were assessed by questionnaire. Medication use was confirmed by a physician interview. Prevalent IHD was identified using the Framingham Heart Study criteria for myocardial infarction and angina pectoris (Shurtleff 1974). Temperature of the room where the electrocardiogram (ECG) was taken was recorded.

### HRV measurement.

We measured HRV between 0600 and 1300 hr using a two-channel (five-lead) ECG monitor (Trillium 3000; Forest Medical, East Syracuse, NY). A detailed description of the HRV measurement protocol is provided elsewhere (Pope et al. 2001). Briefly, after the participants had rested for 5 min, the ECG was recorded (sampling rate of 256 Hz per channel) for approximately 7 min with the subject seated. We used the best 4-consecutive-minute interval for the HRV calculations. The ECG digital recordings were processed, and heart rate and HRV measures were calculated using PC-based software (Trillium 3000 PC Companion Software for MS Windows; Forest Medical). Beats were automatically detected and assigned tentative annotations, which were then reviewed by an experienced scanner to correct for any mislabeled beats or artifacts. Only normal-to-normal (NN) beat intervals were included in the analysis. We computed standard deviation of NN intervals (SDNN), the square root of the mean of the squared differences between adjacent NN intervals (r-MSSD), high-frequency power (HF) (0.15–0.4 Hz), low-frequency power (LF) (0.04–0.15 Hz), and LF:HF ratio. Ninety-two subjects with problematic heart rate measurements (atrial fibrillation, atrial bigeminy and trigeminy, pacemakers, irregular rhythm, irregular sinus rhythm, frequent ventricular ectopic activity, ventricular bigeminy, multifocal atrial tachycardia, or measurement time < 3.5 min) were excluded.

### Air pollution and weather data.

Continuous PM_2.5_, particle number concentration (PN), and black carbon (BC) were measured at the Harvard School of Public Health monitoring site, 1 km from the exam site, using a Tapered Element Oscillating Microbalance (TEOM) (model 1400A; Rupprecht & Pataschnick Co., East Greenbush, NY), condensation particle counter (model 3022A; TSI Inc., Shoreview, MN), and aethalometer (Magee Scientific, Berkeley, CA), respectively. Because the TEOM sample filter is heated to 50°C, a season-specific correction was used to compensate for the loss of semivolatile mass that occurs at this temperature (Allen et al. 1997). Ozone, sulfur dioxide, nitrogen dioxide, carbon monoxide, temperature, and dew-point temperature measurements were obtained from the Massachusetts Department of Environmental Protection local monitoring sites. The gaseous pollutants are measured hourly using U.S. Environmental Protection Agency (EPA) reference methods (U.S. EPA 2002).

To control for weather, we used apparent temperature, defined as a person’s perceived air temperature (O’Neill et al. 2003). It was calculated with the following formula: −2.653 + (0.994 × air temperature) + (0.0153 × dew-point temperature). We estimated missing PM_2.5_, PN, and BC measures using a regression model with date, day of week, hour of day, temperature, relative humidity, pressure, and NO_2_ as predictors (6.4% missing for PM_2.5_, 7.5% for PN, and 0.5% for BC). To evaluate lagged effects of air pollutants, we used 4-hr, 24-hr, and 48-hr moving averages of air pollution matched on the time of measuring ECG for each subject. These averaging times were chosen based on previous reports in the literature.

### Statistical methods.

Measures of HRV were log_10_-transformed to improve normality and stabilize the variance. Linear regression analyses were carried out to evaluate the relation of HRV with each air pollutant. Cardiac medications were categorized as beta-blocker, calcium-channel blocker, and angiotensin-converting enzyme (ACE) inhibitor. After 14 subjects with missing values of the potential confounding factors were excluded, 497 subjects with complete data were available for the analyses.

The following variables were chosen a priori as clinically important predictors and included in the models: age, BMI, fasting blood glucose (FBG), cigarette smoking, use of cardiac medications, room temperature, season, and the lagged moving average of apparent temperature corresponding to the same moving average period for each air pollutant. MAP was also included because this changed the estimated effect of some air pollutants by more than 10%. To model the nonlinear association of apparent temperature with HRV, we used a cubic spline with 3 degrees of freedom (df).

We estimated the percent change in each HRV parameter for 1 SD increase for each pollutant as [10^(β × SD)^ – 1] × 100%, with 95% confidence intervals (CI) {10^[SD × (β± 1.96 × SE)]^ – 1} × 100%, where βand SE are the estimated regression coefficient and its standard error. To test whether observed associations in single-pollutant models were robust to inclusion of another pollutant, two-pollutant models were fitted. To assess modifying effects of hypertension, IHD, diabetes or use of cardiac/antihypertensive medications, we ran separate regressions stratified by those variables and compared the percent changes of each pollutant. We also ran regression models including interaction terms.

## Results

Table 1 shows the demographic and clinical characteristics and HRV measurements of the subjects. The study participants were all male, with average age of 72.7 years (SD = 6.6 years). Seventy-two participants had diabetes (14.5%) on the basis of previously mentioned criteria. Hypertension and IHD prevalences were 67.4 and 28.6%, respectively. People with hypertension were older, had higher levels of BMI and FBG, and were more likely to have IHD, have diabetes, and be taking hypertension medications.

Air pollution and temperature levels are summarized in Table 2. The median time of ECG monitoring was 1000 hr. Levels of all pollutants except O_3_ decreased after peaking around 0700 hr. All air pollution concentrations during the study period were within the National Ambient Air Quality Standards (U.S. EPA 2004).

Of the air pollutants examined, only PM_2.5_ and O_3_ showed several significant associations with the HRV outcomes. Table 3 presents the estimated percent changes of HRV in single- and two-pollutant models for various lags of PM_2.5_ and O_3_. After adjusting for potential confounders, HF decreased by 13.2% (95% CI, –1.0% to 25.4%) and LF:HF ratio increased by 14.5% (95% CI, 2.9–27.5%) per SD (8.0 μg/m^3^) increase in the 24-hr moving average of PM_2.5_. We saw stronger associations with the 48-hr PM_2.5_: a 20.8% (95% CI, 4.6–34.2%) decrease in HF and an 18.6% (95% CI, 4.1–35.2%) increase in LF:HF ratio per 8.0-μg/m^3^ increase. We observed a reduction in LF of 11.5% (95% CI, 0.4–21.3%) associated with 1 SD (13 ppb) increment in the 4-hr O_3_, which was similar in magnitude but only marginally significant with a 24-hr average. In two-pollutant models, the magnitudes of the percent changes for both PM_2.5_ and O_3_ diminished slightly.

We found no significant association of HRV with PN, NO_2_, SO_2_, and CO for any of the exposure averaging periods. For brevity and comparability, Table 4 presents the HRV associations using the averaging periods for gaseous pollutants that showed the strongest effect for O_3_ (4 hr), and the 48-hr averaging period for PN and BC to correspond with the strongest PM_2.5_ effects. An SD (0.47 μg/m^3^) elevation in 48-hr BC was associated with a 13.2% (95% CI, –1.1 to 29.6%) increase in the LF:HF ratio. The point estimates for associations between PN and BC, and HRV measures were negative, but gaseous pollutants (SO_2_ and CO) were positively related.

We also conducted stratified analyses by IHD, hypertension, and diabetes status (Table 5). The associations of all HRV indices with PM_2.5_ and O_3_ were stronger in people with IHD. People with IHD showed 2-fold reductions of SDNN in relation to 48-hr PM_2.5_ compared with people without IHD. The interaction between 4-hr O_3_ and IHD was statistically significant for SDNN (*p* = 0.02 for the interaction term), HF (*p* = 0.01), and LF (*p* = 0.004). We also observed consistently stronger associations between all HRV indices and PM_2.5_ and O_3_ among people with hypertension. The associations observed in SDNN and LF with PM_2.5_ were stronger in people with diabetes, with almost 4-fold higher percent changes. However, diabetes did not modify the effect of O_3_ on HRV.

We assessed whether each antihypertensive medication modified the effects of PM_2.5_ and O_3_ on HRV (Table 6). We found a significant interaction between use of calcium-channel blocker and PM_2.5_ for LF (*p* = 0.04). Moreover, subjects who were not taking a calcium-channel blocker showed larger reductions in SDNN and LF in relation to O_3_. In particular, the association of O_3_ exposure with reduced LF in the full cohort seems to be driven by the subjects not taking calcium-channel blockers, with a substantial (although imprecisely estimated) increase in LF associated with O_3_ exposure in subjects on the drug. As a result of this effect on LF as well as HF, a marginally significant association was seen between O_3_ and SDNN (total HRV) as well.

We found no significant interaction between PM_2.5_ and O_3,_ and use of beta-blocker or ACE inhibitor. However, the effect of both pollutants on LF was substantially reduced by beta-blocker drugs. In those taking beta-blockers, the decrease in HF was larger than that in LF in relation to PM_2.5_, compared with those who had never taken those medications. Thus, a larger increase in LF:HF ratio was observed in participants who were taking beta-blocker. However, the association with ACE inhibitors was opposite: There was a larger increase in LF:HF ratio associated with PM_2.5_ among those not taking that medication.

## Discussion

This study is consistent with previous evidence that PM and O_3_ are associated with decreased HRV, particularly for PM and HF, a parasympathetic (vagal) modulation of the heart. The associations of HRV were strongest with the 48-hr moving averages of particles, but O_3_ had a shorter-term impact (4 hr and 24 hr). Furthermore, subjects with IHD and hypertension appeared to have larger reductions in HRV measures in relation to both PM_2.5_ and O_3_ exposures. People with diabetes had larger decreases of SDNN and LF associated with PM_2.5_. In addition, we found evidence for an association with BC, a marker of traffic particles.

When we examined medications, calcium-channel blockers had the most profound effect on the pollution associations, particularly for O_3_. This modification was primarily on LF, suggesting that this drug is blocking effects of pollution on the sympathetic pathway. If anything, the parasympathetic response was enhanced in these subjects. As expected, beta-blockers seemed to reduce the LF response of both pollutants. By contrast, use of ACE inhibitors did not consistently or significantly modify pollution effects. Drug use patterns in these subgroups are related to underlying conditions, thus limiting the interpretability of these results. Nevertheless, they suggest that air pollution has the ability to affect both sympathetic and parasympathetic pathways. The sympathetic response seems mediated by pathways related to calcium flux into cells, whereas the parasympathetic response seems likely to be due to other mechanisms.

Previous studies have consistently reported PM associations with decreased HRV in older adults (Creason et al. 2001; Gold et al. 2000; Holguin et al. 2003; Liao et al. 1999, 2004; Pope et al. 1999, 2004) (Table 7). Our results are consistent with those. For example, estimated decreases in HF resulting from an exposure to a PM_2.5_ increment of 10 μg/m^3^ in the previous studies were 24.1, 14.9, 19.3, and 5.1%. The last result is for a 10-μg/m^3^ increase in PM_10_ and is not directly comparable. We found a 16.2% reduction. Given the CIs (Table 7), these look fairly similar.

Three studies also evaluated the effect of O_3_ on cardiac autonomic function, primarily HF (Gold et al. 2000; Holguin et al. 2003; Liao et al. 2004). The difference in measuring times used in the studies preclude quantitative comparisons of results, but there was substantial variability. In the present study, O_3_-related decreases in HF ranged from 2.6 to 11.1% depending on choice of moving averages of O_3_, but all estimates were insignificant.

Current knowledge about pathophysiologic mechanisms that connect air pollution exposure and alterations in the autonomic nervous system is limited. One plausible mechanism is that inhalation of PM causes oxidative stress directly or via acute pulmonary inflammation. Oxidative stress in the lungs seems to induce proinflammatory mediators, such as cytokines (Donaldson et al. 2001), to increase extracellular calcium influx possibly through activation of calcium channels in the plasma membrane (Stone et al. 2000), and to inactivate nitric oxide (Thomas et al. 2001). These effects are considered to cause an increase in sympathetic and a reduction in vagal tone (Aronson et al. 2001; Chowdhary et al. 2002; Rodenbaugh et al. 2003), which may be linked with cardiac events, such as ventricular arrhythmias and myocardial infarction. In general, we find air pollution associated with greater reductions in vagal tone than in sympathetic activity.

A study from the Utah Valley (USA) found positive associations between PM_10_ and r-MSSD (Pope et al. 1999). Additionally, dogs exposed to concentrated ambient air particles showed significantly higher HF and LF compared with filtered air exposure (Godleski et al. 2000). Godleski et al. (2000) argued that too much elevation in parasympathetic stimulation may deteriorate cardiac status and result in a fatal bradyarrhythmia. A large follow-up study in Rotterdam, the Netherlands, found that elderly subjects in the highest quartile as well as the lowest quartile of SDNN had significantly increased risks for cardiac mortality, suggesting that in the elderly, alterations in HRV in either direction might be adverse (de Bruyne et al. 1999).

Few previous studies have evaluated modifiers of the air pollution–HRV association. Our results agree with two such studies, which showed larger decreases in HRV among people with hypertension (Holguin et al. 2003; Liao et al. 2004). Although dysregulation of the autonomic nervous system plays a role in the pathogenesis of hypertension, the causal mechanism of modification by hypertension has not been discussed. Hypertension is associated with lower baseline HRV and endothelial dysfunction (John and Schmieder 2003; Schroeder et al. 2003; Singh et al. 1998). Hypertensive people may have higher levels of oxidative-stress–induced inflammatory responses. These existing impairments may make hypertensive people less able to accommodate the additional oxidative stress related to air pollution exposure and therefore could explain the enhanced effect on HRV.

We observed a larger reduction in HRV among people with diabetes compared with subjects without diabetes. Diabetes is known to be associated with low autonomic function (Burger and Aronson 2001; Singh et al. 2000), and has been reported to modify the association of PM with both hospital admissions (Zanobetti and Schwartz 2001) and deaths (Bateson and Schwartz 2004). Several epidemiologic studies showed that LF power, which reflects mainly sympathetic modulation, was more influenced by diabetes than any other HRV index (Burger and Aronson 2001; Singh et al. 2000). The present study also showed that decreases in LF in relation to PM_2.5_ exposure were larger in people with diabetes than those in people without diabetes (−19.1 vs. −5.0%). Both diabetes and PM have been associated with oxidative-stress–induced inflammation and endothelial and autonomic dysfunctions. Therefore, susceptible individuals who have preexisting inflammation due to diabetes may be more responsive to airborne particles exposure.

We found the strongest effects of PM_2.5_ and O_3_ in 48-hr and 4-hr moving averages, respectively. The rationale for the moving average model is that air pollution can lead to adverse health events occurring not only on the same day but also on several subsequent days (Schwartz 2000). Hence, the response to an acute pollution exposure could be distributed over a number of days. Because hourly measured concentrations of air pollution were available, we could evaluate several lagged models with end times matched to each participant’s ECG measure, an improvement over traditional approaches using fixed calendar days. We found stronger particle pollutant associations in longer lagged models but stronger O_3_ associations in shorter ones.

A potential limitation of this study is that we measured ECG once for each subject, so subject-specific variation of HRV measures may not be ruled out as a potential confounder. However, this variation would have to be correlated with air pollution levels for it to confound the observed associations. We collected information on many possible factors that would affect autonomic function, but the covariates included in the model may not cover all predictors of individual variations of HRV. A longitudinal design would provide for better adjustment of within-subject variation in the observed associations and allow examination of differences in baseline autonomic function over time.

In this study, many potential confounding factors were included in the model. BMI; blood total cholesterol, high-density lipoprotein, and triglyceride levels; alcohol consumption; and respiratory and cardiovascular disease history did not confound the association between air pollution and HRV. We also measured the ECG at a stable temperature and adjusted for the temperature of the room where the ECG was taken, as well as for ambient meteorologic factors, including apparent temperature and season. Therefore, the observed associations are less likely to reflect bias due to the confounding factors.

Although we did not conduct personal exposure monitoring during the time of the test, the monitoring site was relatively close (1 km) to the examination site. Moreover, evidence suggests that ambient measures of PM have relatively uniform spatial distribution across urban areas and the longitudinal correlation between daily changes in exposure and daily changes in ambient concentrations are high (Sarnat et al. 2000). Therefore, PM concentrations at the monitoring site should be a good surrogate of PM exposure.

This study cohort consists of all males and almost all whites. Sex and race may be important determinants of HRV as well as modifiers of the association between air pollution and HRV, as was observed by Liao et al. (2004).

This population-based study suggests that short-term exposures to PM_2.5_ and O_3_ are predictors of alterations in cardiac autonomic function as measured by HRV among older adults. Persons with IHD, hypertension, and diabetes appear to be more susceptible to autonomic dysfunction related to PM_2.5_ exposure. The consistency of the effect modification observed in this and other studies strengthens evidence that these conditions mark susceptibility to air pollution exposure and provides new information to guide research on underlying biologic mechanisms.

## Figures and Tables

**Table 1:** Characteristics [mean ± SD or *n* (%)] of the study subjects.

		Hypertension	
Variable	All subjects (*n* = 497)	Without (*n* = 162)	With (*n* = 335)
Age (years)	72.7 ± 6.6	71.2 ± 6.5	73.4 ± 6.5
BMI (kg/m^2^)	28.3 ± 4.1	27.2 ± 3.9	28.7 ± 4.1
Systolic blood pressure (mm Hg)	131.4 ± 16.3	125.0 ± 10.5	134.5 ± 17.7
Diastolic blood pressure (mm Hg)	75.7 ± 9.4	75.3 ± 7.1	75.9 ± 10.3
MAP (mm Hg)	94.3 ± 10.2	91.9 ± 7.2	95.5 ± 11.2
Heart rate (beat/min)	70.7 ± 6.7	71.4 ± 5.8	70.4 ± 7.1
Fasting blood glucose (mg/dL)	108.0 ± 29.0	103.1 ± 22.3	110.3 ± 31.5
Cholesterol (mg/dL)	197.0 ± 37.6	207.1 ± 36.2	192.1 ± 37.4
High-density lipoprotein (mg/dL)	49.7 ± 13.5	52.9 ± 15.3	48.1 ± 12.3
Triglycerides (mg/dL)	129.8 ± 71.5	122.0 ± 66.7	133.5 ± 73.5
Smoking status [*n* (%)]			
Never smoker	160 (32.2)	58 (35.8)	102 (30.4)
Former smoker	311 (62.6)	93 (57.4)	218 (65.1)
Current smoker	26 (5.2)	11 (6.8)	15 (4.5)
Alcohol intake (≥2 drinks/day) [*n* (%)]	96 (19.3)	30 (18.5)	66 (19.7)
Diabetes mellitus [*n* (%)]	72 (14.5)	14 (8.6)	58 (17.3)
IHD history [*n* (%)]	142 (28.6)	16 (9.9)	126 (37.6)
Use of beta-blocker [*n* (%)]	163 (32.8)	0 (0.0)	163 (48.7)
Use of calcium-channel blocker [*n* (%)]	70 (14.1)	0 (0.0)	70 (20.9)
Use of ACE inhibitor [*n* (%)]	100 (20.1)	0 (0.0)	100 (29.9)
HRV			
Log_10_ SDNN, msec	1.5 ± 0.25	1.5 ± 0.25	1.5 ± 0.25
Log_10_ HF, msec^2^	1.9 ± 0.66	1.8 ± 0.62	1.9 ± 0.68
Log_10_ LF, msec^2^	2.0 ± 0.52	2.0 ± 0.50	2.0 ± 0.54
Log_10_ LF:HF	0.10 ± 0.49	0.22 ± 0.47	0.04 ± 0.49

**Table 2:** Twenty-four–hour moving averages of outdoor air pollution and apparent temperature, and room temperature during the HRV measurement.

	Mean ± SD	Range
PM_2.5_ (μg/m^3^)	11.4 ± 8.0	0.45–62.9
PN concentration (no./cm^3^)	28,942 ± 13,527	8,538–74,675
BC (μg/m^3^)	0.92 ± 0.47	0.19–2.6
O_3_ (ppb)	23.0 ± 13.0	2.6–84.5
NO_2_ (ppb)	22.7 ± 6.2	7.0–40.1
SO_2_ (ppb)	4.9 ± 3.4	0.95–24.7
CO (ppm)	0.50 ± 0.24	0.13–1.8
Apparent temperature (°C)	11.4 ± 9.9	−5.2–35.6
Room temperature (°C)	24.5 ± 1.4	20.0–30.0

**Table 3:** Estimated percent changes (95% CIs) in HRV in single (PM_2.5_ or O_3_) and two-pollutant (PM_2.5_ and O_3_) models for PM_2.5_ and O_3_ in various lagged moving averages.

Outcome, model, predictor	4-hr moving average	24-hr moving average	48-hr moving average
Log_10_ SDNN			
Single-pollutant			
PM_2.5_	−0.1 (−5.0 to 4.9)	−2.2 (−7.7 to 3.6)	−5.4 (−11.8 to 1.5)
O_3_	−3.6 (−8.9 to 2.0)	−5.3 (−11.7 to 1.7)	−2.2 (−10.0 to 6.1)
Two-pollutant			
PM_2.5_	0.2 (−4.8 to 5.5)	−0.3 (−6.6 to 6.3)	−5.0 (−12.2 to 2.7)
O_3_	−3.6 (−9.0 to 2.1)	−5.1 (−12.2 to 2.5)	−0.2 (−8.8 to 9.1)
Log_10_ HF			
Single-pollutant			
PM_2.5_	−6.3 (−17.8 to 6.7)	−13.2 (−25.4 to 1.0)*	−20.8 (−34.2 to −4.6)**
O_3_	−9.3 (−21.8 to 5.3)	−11.1 (−26.2 to 7.1)	−2.6 (−21.6 to 21.1)
Two-pollutant			
PM_2.5_	−5.1 (−17.1 to 8.6)	−8.6 (−22.9 to 8.3)	−20.3 (−35.2 to −2.1)**
O_3_	−9.4 (−22.1 to 5.4)	−7.9 (−24.9 to 13.0)	6.5 (−15.9 to 34.9)
Log_10_ LF			
Single-pollutant			
PM_2.5_	5.7 (−4.6 to 17.1)	−0.6 (−11.9 to 12.1)	−6.0 (−18.9 to 8.9)
O_3_	−11.5 (−21.3 to −0.4)**	−10.9 (−23.1 to 3.3)	−6.3 (−21.1 to 11.2)
Two-pollutant			
PM_2.5_	6.2 (−4.6 to 18.1)	3.9 (−9.2 to 18.8)	−3.6 (−18.1 to 13.5)
O_3_	−11.3 (−21.3 to −0.1)**	−12.2 (−25.3 to 3.2)	−5.0 (−21.2 to 14.6)
Log_10_ (LF:HF)			
Single-pollutant			
PM_2.5_	12.9 (3.0 to 23.7)**	14.5 (2.9 to 27.5)**	18.6 (4.1 to 35.2)**
O_3_	−2.4 (−12.1 to 8.3)	0.2 (−12.1 to 14.2)	−3.9 (−17.4 to 11.9)
Two-pollutant			
PM_2.5_	11.9 (1.8 to 22.9)**	13.7 (0.9 to 28.0)**	21.0 (4.8 to 39.8)**
O_3_	−2.1 (−12.0 to 8.8)	−4.7 (−17.4 to 9.9)	−10.7 (−24.4 to 5.3)

Coefficients are expressed as percent change per 1 SD (8 μg/m^3^ for PM_2.5_ and 13 ppb for O_3_), adjusting for age; BMI; MAP; FBG; cigarette smoking; use of beta-blocker, calcium-channel blocker, and/or ACE inhibitor; room temperature; season; and cubic smoothing splines (3 df) for moving averages of apparent temperature corresponding for the predictor.

* *p* < 0.1.

** *p* < 0.05.

**Table 4:** Estimated percent changes (95% CIs) in HRV for other pollutants.

	48-hr moving average		4-hr moving average		
Outcome	PN	BC	NO_2_	SO_2_	CO
Log_10_ SDNN	−0.7 (−9.3 to 8.9)	−3.4 (−10.2 to 3.9)	1.2 (−3.1 to 5.7)	2.3 (−1.7 to 6.4)	2.0 (−2.9 to 7.3)
Log_10_ HF	−4.1 (−24.7 to 22.1)	−13.8 (−28.9 to 4.4)	−0.9 (−11.7 to 11.2)	5.6 (−4.9 to 17.3)	8.8 (−4.6 to 24.1)
Log_10_ LF	−7.0 (−23.2 to 12.6)	−2.4 (−16.2 to 13.6)	1.1 (−7.7 to 10.7)	2.2 (−5.9 to 11.1)	3.2 (−7.0 to 14.6)
Log_10_ (LF:HF)	−3.0 (−18.2 to 15.0)	13.2 (−1.1 to 29.6)*	2.0 (−5.9 to 10.6)	−3.2 (−10.1 to 4.2)	−5.1 (−13.5 to 4.1)

Coefficients are expressed as percent change per 1 SD (13,527/cm^3^ for PN, 0.47 μg/m^3^ for BC, 6.2 ppb for NO_2_, 3.4 ppb for SO_2_, and 0.24 ppm for CO) adjusting for age; BMI; MAP; FBG; cigarette smoking; use of beta-blocker, calcium-channel blocker, and/or ACE inhibitor; room temperature; season; and cubic smoothing splines (3 df) for moving averages of apparent temperature corresponding for the predictor.

* *p* < 0.1.

**Table 5:** Estimated percent changes (95% CIs) in HRV associated with 48-hr PM_2.5_ and 4-hr O_3_ stratified by hypertension, IHD, and diabetes.

	Hypertension		IHD		Diabetes	
Outcome, predictor	Without (*n* = 162)*^a^*	With (*n* = 335)*^a^*	Without (*n* = 355)	With (*n* = 142)	Without (*n* = 425)*^b^*	With (*n* = 72)*^b^*
Log_10_ SDNN						
PM_2.5_	−2.4 (−13.2 to 9.8)	−8.1 (−15.7 to 0.3)*	−3.5 (−11.1 to 4.7)	−8.4 (−20.4 to 5.5)	−4.7 (−11.4 to 2.6)	−16.6 (−36.3 to 9.2)
O_3_	1.8 (−7.4 to 11.8)	−5.5 (−12.1 to 1.5)*	−1.0 (−7.3 to 5.8)	−8.1 (−17.7 to 2.7)	−5.7 (−11.1 to 0.1)*	4.0 (−17.3 to 30.6)
Log_10_ HF						
PM_2.5_	−14.4 (−36.8 to 15.9)	−24.5 (−40.4 to −4.5)**	−18.0 (−33.7 to 1.5)*	−24.1 (−48.3 to 11.4)	−20.8 (−34.8 to −3.9)**	−17.0 (−58.3 to 65.1)
O_3_	8.8 (−14.7 to 38.7)	−17.0 (−31.6 to 0.7)*	1.6 (−14.4 to 20.5)	−29.4 (−47.6 to −4.9)**	−13.7 (−26.3 to 1.0)*	5.7 (−40.7 to 88.1)
Log_10_ LF						
PM_2.5_	−2.9 (−23.5 to 23.2)	−10.5 (−25.8 to 7.9)	−7.0 (−21.3 to 9.9)	0.5 (−26.7 to 37.7)	−5.0 (−18.6 to 10.8)	−19.1 (−54.2 to 42.9)
O_3_	−5.4 (−21.6 to 14.1)	−12.6 (−25.0 to 1.9)*	−4.8 (−16.7 to 8.8)	−25.8 (−41.9 to −5.3)**	−13.2 (−23.4 to −1.7)**	−8.4 (−41.7 to 44.1)
Log_10_ LF:HF						
PM_2.5_	13.5 (−9.4 to 42.1)	18.6 (0.4 to 40.0)**	13.3 (−2.4 to 31.6)	32.5 (0.5 to 74.7)*	20.0 (4.8 to 37.5)**	−2.6 (−39.4 to 56.6)
O_3_	−13.1 (−27.0 to 3.5)	5.3 (−8.2 to 20.8)	−6.3 (−16.7 to 5.5)	5.1 (−15.9 to 31.4)	0.6 (−9.9 to 12.4)	−13.3 (−41.1 to 27.6)

Coefficients are expressed as percent change per 1 SD (8 μg/m^3^ for PM_2.5_ and 13 ppb for O_3_) adjusting for age; BMI; MAP; FBG; cigarette smoking; use of beta-blocker, calcium-channel blocker, and/or ACE inhibitor; room temperature; season; and cubic smoothing splines (3 df) for moving averages of apparent temperature corresponding for the predictor.

a MAP and use of beta-blocker, calcium-channel blocker, and/or ACE inhibitor not included in the model.

b FBG not included in the model.

* *p* < 0.1.

** *p* < 0.05.

**Table 6:** Estimated percent changes (95% CIs) in HRV associated with 48-hr PM_2.5_ and 4-hr O_3_ stratified by use of beta-blocker, calcium-channel blocker, and ACE inhibitor.

	Use of beta-blocker		Use of calcium channel blocker		Use of ACE inhibitor	
Outcome, predictor	No (*n* = 334)*^a^*	Yes (*n* = 163)*^a^*	No (*n* = 427)*^b^*	Yes (*n* = 70)*^b^*	No (*n* = 397)*^c^*	Yes (*n* = 100)*^c^*
Log_10_ SDNN						
PM_2.5_	−4.6 (−12.4 to 4.0)	−7.5 (−18.5 to 5.1)	−6.9 (−13.8 to 0.6)*	2.8 (−14.3 to 23.3)	−5.1 (−12.0 to 2.4)	−1.8 (−19.4 to 19.6)
O_3_	−5.0 (−11.2 to 1.6)	−0.5 (−11.1 to 11.4)	−4.9 (−10.6 to 1.3)	1.2 (−11.7 to 16.1)	−2.8 (−8.7 to 3.4)	−9.2 (−21.9 to 5.5)
Log_10_ HF						
PM_2.5_	−17.8 (−34.2 to 2.6)*	−25.4 (−47.3 to 5.7)	−23.1 (−37.1 to −6.1)**	−15.6 (−49.5 to 41.2)	−23.5 (−37.4 to −6.5)**	8.7 (−34.2 to 79.7)
O_3_	−7.0 (−21.7 to 10.6)	−14.8 (−37.1 to 15.5)	−10.9 (−24.2 to 4.8)	−9.7 (−38.4 to 32.4)	−7.0 (−21.0 to 9.6)	−23.7 (−48.2 to 12.4)
Log_10_ LF						
PM_2.5_	−11.3 (−25.3 to 5.4)	−0.5 (−25.0 to 32.0)	−9.3 (−22.4 to 6.0)	−0.6 (−36.5 to 55.8)	−6.0 (−19.6 to 9.8)	8.8 (−29.4 to 67.7)
O_3_	−13.8 (−24.5 to −1.5)**	−8.1 (−28.3 to 17.8)	−14.4 (−24.5 to −2.9)**	11.6 (−19.8 to 55.4)	−11.3 (−21.8 to 0.6)*	−12.3 (−37.0 to 22.2)
Log_10_ LF:HF						
PM_2.5_	8.0 (−8.2 to 27.0)	33.4 (6.5 to 67.0)**	17.9 (2.5 to 35.7)**	17.8 (−19.0 to 71.4)	22.8 (6.7 to 41.3)**	0.1 (−30.1 to 43.5)
O_3_	−7.3 (−18.2 to 5.0)	7.8 (−11.5 to 31.3)	−3.9 (−14.2 to 7.6)	23.5 (−5.8 to 62.0)	−4.7 (−15.0 to 6.8)	15.0 (−13.1 to 52.3)

Coefficients are expressed as percent change per 1 SD (8 μg/m^3^ for PM_2.5_ and 13 ppb for O_3_) adjusting for age; BMI; MAP; FBG; cigarette smoking; use of beta-blocker, calcium-channel blocker, and/or ACE inhibitor; room temperature; season; and cubic smoothing splines (3 df) for moving averages of apparent temperature corresponding for the predictor.

a Use of beta-blocker not included in the model.

b Use of calcium-channel blocker not included in the model.

c Use of ACE inhibitor not included in the model.

* *p* < 0.1.

** *p* < 0.05.

**Table 7:** Summary of the studies that assessed the association between ambient PM and HRV.

Reference	Design	Population (no./mean or age range/study area)	Ambient PM level (μg/m^3^)	Covariates adjusted	Main results*^a^*
Liao et al. 1999	Longitudinal	26 volunteers (3 weeks) Mean, 81 years Baltimore	24-hr PM_2.5_, 16.1 ± 6.9	Age, sex, cardiovascular health status	SDNN, −8.8 (−14.9 to 0.0) HF, −24.1 (−42.5 to 0.0) LF, −22.4 (−39.7 to 0.0)
Pope et al. 1999	Longitudinal	7 (29 person days) Mean, 70 years Utah Valley	PM_10_, no concentration reported	Barometric pressure at 1700 hr mountain time, HR	SDNN, −1.4 (−2.1 to −0.6) SDANN, −1.4 (−2.4 to −0.5) r-MSSD, 1.9 (−0.2 to 3.9)
Gold et al. 2000	Longitudinal	21 (163 observation) Range, 53−87 years Boston	4-hr PM_2.5_, 15.3 (Range, 2.9–48.6)	Uses of calcium or beta-blockers, ACE inhibitor	At slow breathing: SDNN, −2.9 (−7.8 to 2.1) r-MSSD, −10.6 (−18.3 to −2.9)
Creason et al. 2001	Longitudinal	56 nonsmokers (4 weeks) Mean, 82 years Baltimore	24-hr PM_2.5_, 20.5 (Range, 7.8–45.3)	Age, sex, CV status, trend, maximum temperature, mean DPT	HF, −14.9 (−25.9 to −4.5) LF, −12.9 (−20.6 to −2.3)
Holguin et al. 2003	Longitudinal	34 (384 observations) Mean, 79 years Mexico City	24-hr PM_2.5_, 30.4 ± 9.9	Age, HR, hypertension	HF, −19.3 (−29.2 to −7.7) LF, −8.4 (−19.3 to 0.2) LF:HF, 24.2 (−7.5 to 66.7)
Pope et al. 2004	Longitudinal	88 (250 observations) Range, 54–89 years Utah	24-hr PM_2.5_, 23.7 ± 20.2	Interactive spline smooths for temperature, RH, HR	SDNN, −2.7 (−3.9 to −1.4) SDANN, −1.7 (−3.3 to −0.2) r-MSSD, −6.1 (−9.2 to −3.0)
Liao et al. 2004	Cross-sectional	4,899 Mean, 62 years ARIC Study	24-hr PM_10_, 24.3 ± 11.5	Age, sex, ethnicity, BMI, education, smoking, CV medications, CHD, diabetes, hypertension, HR, season, temperature, RH, sky cover	SDNN, −2.4 (−3.8 to −1.0) HF, −5.1 (−8.0 to −2.1) LF, −1.7 (−4.7 to 1.3)
Present study	Cross-sectional	497 males Mean, 73 years Normative Aging Study in Boston	24-hr PM_2.5_, 11.4 ± 8.0	Age, MAP, smoking, FBG, use of ACE inhibitor, room temperature, apparent temperature, season	SDNN, −2.7 (−9.5 to 4.6) HF, −16.2 (−30.7 to 1.3) LF, −0.7 (−14.6 to 15.4) LF:HF, 18.5 (3.7 to 35.4)

Abbreviations: SDANN, standard deviation of all 5-min NN interval means; CHD, coronary heart disease; CV, cardiovascular disease; HR, heart rate; DPT, dew-point temperature; RH, relative humidity.

a Percent change (95% CI) for an increase of 10 μg/m^3^ in PM_2.5_.
